# Epidemiological Characteristics and Prognostic Scoring in Toxic Epidermal Necrolysis and Stevens–Johnson Syndrome: Insights from a 17-Year Burn Center Experience

**DOI:** 10.3390/medicina61010066

**Published:** 2025-01-03

**Authors:** David Breidung, Sarina Delavari, Ioannis-Fivos Megas, Alexander Geierlehner, Wolfgang Hitzl, Karl J. Bodenschatz, Konrad Karcz, Denis Ehrl, Moritz Billner

**Affiliations:** 1 Department of Plastic, Reconstructive and Hand Surgery, Burn Unit, Klinikum Nuremberg Hospital, Paracelsus Medical University, Breslauer Str. 201, 90471 Nuremberg, Germany; sarina.delavari@klinikum-nuernberg.de (S.D.); ioannis.megas@jsd.de (I.-F.M.); alexander.geierlehner@uk-erlangen.de (A.G.); konrad.karcz@klinikum-nuernberg.de (K.K.); denis.ehrl@klinikum-nuernberg.de (D.E.); moritz.billner@klinikum-nuernberg.de (M.B.); 2 Department of Orthopaedic and Trauma Surgery, Center of Plastic Surgery, Hand Surgery and Microsurgery, Evangelisches Waldkrankenhaus Spandau, Stadtrandstraße 555, 13589 Berlin, Germany; 3 Department of Plastic and Hand Surgery and Laboratory for Tissue Engineering and Regenerative Medicine, University Hospital Erlangen, Friedrich Alexander University Erlangen-Nürnberg (FAU), Krankenhausstrasse 12, 91054 Erlangen, Germany; 4 Research and Innovation Management (RIM), Paracelsus Medical University Salzburg, 5020 Salzburg, Austria; wolfgang.hitzl@pmu.ac.at; 5 Department of Ophthalmology and Optometry, Paracelsus Medical University Salzburg, Muellner Hauptstr. 48, 5020 Salzburg, Austria; 6 Research Program Experimental Ophthalmology and Glaucoma Research, Paracelsus Medical University, Muellner Hauptstr. 48, 5020 Salzburg, Austria; 7 Department for Pediatric Surgery, Nuremberg Hospital, Paracelsus Medical University, Breslauer Str. 201, Prof.-Ernst-Nathan Straße 1, 90419 Nuremberg, Germany; karl.bodenschatz@klinikum-nuernberg.de

**Keywords:** toxic epidermal necrolysis, Stevens–Johnson syndrome, drug reaction, SCORTEN, ABCD-10

## Abstract

*Background and Objectives*: Toxic epidermal necrolysis (TEN) and Stevens–Johnson syndrome (SJS) are rare yet life-threatening dermatologic conditions characterized by severe skin and mucous membrane involvement. Accurate prognostic systems are crucial for clinical management to assess disease severity and predict outcomes. The primary objective of this study was to assess the epidemiological characteristics and clinical outcomes of patients with Stevens–Johnson syndrome (SJS), toxic epidermal necrolysis (TEN), and SJS/TEN overlap over a 17-year period at a specialized burn center. The secondary objectives were to evaluate the performance of existing prognostic scoring systems (SCORTEN, Re-SCORTEN, and ABCD-10) in predicting mortality and to propose a novel classification tree model to improve mortality prediction. *Materials and Methods*: A 17-year retrospective study at a burn center included 68 patients with SJS, SJS/TEN overlap, or TEN. Demographic, clinical, laboratory data, and prognostic scores (SCORTEN, Re-SCORTEN, ABCD-10) were collected and analyzed for associations with mortality. A classification tree was created to detect unknown determinants of SJS/TEN mortality. *Results*: The drug most frequently associated with the occurrence of SJS/TEN was metamizole. The mortality rate was 51%. Affected body surface area, platelet count, and serum blood urea nitrogen differed significantly between survivors and non-survivors. Regarding the scoring systems, only the Re-SCORTEN showed reliable differentiation for these groups. A classification tree model achieved an accuracy of 89% in predicting the mortality risk. In the ROC curve analysis, the AUC values were 0.88 for the classification tree, 0.66 for Re-SCORTEN, 0.61 for SCORTEN, and 0.56 for ABCD-10. *Conclusions*: This study explores mortality predictors in SJS/TEN via a classification tree model, highlighting potential factors for further investigation. While cautioning against immediate clinical application due to data constraints, the findings underscore the need for larger studies to validate and refine prediction models in this context.

## 1. Introduction

Toxic epidermal necrolysis (TEN) and Stevens–Johnson syndrome (SJS) represent rare but life-threatening dermatologic conditions characterized by severe skin and mucous membrane involvement [1,2,3]. These disorders, often triggered by adverse drug reactions or infections [4], lead to a cascade of cutaneous and systemic reactions, necessitating prompt and intensive medical intervention. Differentiation between SJS, TEN, and SJS/TEN overlap can be made on the basis of the extent of detachment of the body surface area [1,2]. Below 10% of the body surface area the condition is referred to as SJS, between 10 and 30% as SJS/TEN overlap, and above 30% as TEN [1,2]. In this study, the term SJS/TEN is used when referring generally to SJS, TEN, and/or SJS/TEN overlap.

These conditions begin usually with flu-like symptoms such as fever, sore throat, and conjunctivitis, followed by the sudden appearance of erythematous macules that coalesce into blisters and detach, leading to large sheets of epidermal loss [1,3]. Mucosal involvement is seen in over 90% of cases, affecting the eyes, oral cavity, and genital mucosa, resulting in conjunctivitis, oral erosions, and genital ulcers [2]. A positive Nikolsky sign, where slight pressure causes epidermal separation, is a key diagnostic feature [1,2,5]. In addition to cutaneous involvement, systemic complications can occur, affecting the respiratory, gastrointestinal, and urinary tracts [3]. Ocular complications are among the most severe long-term sequelae, with conditions such as conjunctival synechiae, corneal ulceration, and chronic dry eye often requiring long-term ophthalmological care [2]. Survivors may experience chronic pigmentary changes, nail dystrophy, and strictures in the gastrointestinal, ocular, and genital mucosa [2,3].

The treatment of SJS/TEN requires immediate discontinuation of the causative drug, supportive care, and the use of immunomodulatory therapies when appropriate. Early identification and withdrawal of the culprit drug is the most critical step in reducing mortality [1,2]. Supportive care includes fluid and electrolyte replacement, nutritional support, temperature regulation, and infection prevention, often requiring care in specialized burn units [1,2,3]. Non-adhesive dressings are used for wound care to avoid further epidermal detachment, while active debridement is generally avoided [1,3,6]. Pharmacological therapies such as intravenous immunoglobulin (IVIG) have been used to inhibit Fas–FasL-mediated keratinocyte apoptosis, although the evidence for its efficacy remains inconclusive [2,3]. Cyclosporin (CsA) has shown promising results in halting disease progression and promoting faster re-epithelialization [2]. Corticosteroids are controversial due to the risk of secondary infections, but in some cases, early high-dose therapy has shown potential benefits [1,2]. Anti-TNF agents, such as infliximab, have been reported to stop disease progression in individual cases [3]. Plasma exchange has been proposed to remove circulating immune complexes, but current evidence supporting its effectiveness is limited [3]. Ocular care is essential to prevent long-term complications like conjunctival synechiae and corneal scarring, which may require urgent ophthalmologic intervention [2]. Optimal management of SJS/TEN relies on a multi-disciplinary approach involving dermatology, ophthalmology, and intensive care specialists to reduce both mortality and long-term sequelae [2,3].

Over the years, the understanding and management of SJS and TEN has evolved, with advances in medical care and treatment protocols aimed at improving patient outcomes [7]. Prognostic systems for SJS/TEN play a critical role in clinical management by assisting healthcare professionals in assessing the severity of the disease and predicting patient outcomes [8]. These systems consider various clinical and laboratory parameters and should allow risk-stratifying patients at admission [9]. Consequently, prognostic scoring systems could facilitate timely intervention and ultimately improve survival and minimize long-term complications in affected patients.

The prognostic scores and their parameters are shown in Table 1. In 2000, Bastuji-Garin et al. presented SCORTEN, a Severity-of-Illness Score for toxic epidermal necrolysis, which classifies patients with SJS, TEN, and SJS/TEN overlap into five risk groups based on their mortality risk using seven parameters [10]. Noe et al. 2019 introduced the ABCD-10 score with data from a multicenter study, which assigns a predicted mortality rate to patients with SJS/TEN based on four parameters [11]. An extension of SCORTEN was presented by Koh et al. in 2022 with Re-SCORTEN, which proposes the red cell distribution (RDW)/hemoglobin (Hb) ratio as an additional criterion [12]. Of the prognostic scores, SCORTEN represents the best known score to date [9]. In a meta-analysis by Torres-Navarro et al., SCORTEN exhibited reasonable predictive accuracy for mortality [8]. However, their findings suggested that the influence of various factors, including comorbidities, may warrant a reconsideration of the scale’s criteria.

In this study, we delve into a 17-year experience from a burn center, where cases of SJS, TEN, and SJS/TEN overlap have been meticulously documented and treated. The primary aim of this study was to explore the epidemiological characteristics and clinical outcomes of patients with SJS, TEN, and SJS/TEN overlap who were treated at our burn center over a 17-year period. The secondary aims were (1) to evaluate the predictive performance of established prognostic scoring systems (SCORTEN, Re-SCORTEN, and ABCD-10) for mortality in SJS/TEN patients and (2) to develop and present a novel classification tree model to enhance the prediction of mortality in this patient group. Through this endeavor, we aspire to enhance the clinical management of SJS/TEN, ultimately improving the outcomes and quality of life for affected individuals.

## 2. Materials and Methods

We conducted a retrospective study at the Department of Plastic, Reconstructive and Hand Surgery, Burn Unit at Klinikum Nuremberg Hospital, Paracelsus Medical University. This study included patients with a clinical diagnosis of Stevens–Johnson syndrome (SJS), toxic epidermal necrolysis (TEN), or SJS/TEN overlap who were treated at the burn unit of our hospital between January 2006 and July 2023. To ensure diagnostic accuracy, only patients whose diagnosis was confirmed through histopathological examination of skin biopsies were included in the analysis. Eligible patients were required to have complete clinical and laboratory data, as well as available information on mortality outcomes.

Patients were excluded from the study if they did not have histopathological confirmation of SJS/TEN, as clinical diagnosis alone was deemed insufficient for inclusion. Furthermore, patients with incomplete or missing clinical or laboratory data that would preclude meaningful analysis were excluded. Additionally, patients who were transferred from other institutions and received treatment outside the burn unit for the majority of their hospital stay were not included in the study cohort. These exclusion criteria were applied to ensure the homogeneity and reliability of the study population. A total of 68 patients met all inclusion criteria and were included in the final analysis. This well-defined cohort allowed for a robust evaluation of epidemiological characteristics, clinical predictors, and the performance of prognostic scoring systems in SJS/TEN.

Patients were identified using the corresponding ICD codes L51.20 (with involvement of less than 30% of the body surface) and L51.21 (with involvement of 30% or more of the body surface) from our database. Admission reports, discharge reports, operative reports, and nursing charts, as well as field reports, were consulted for data collection. Demographic data, data regarding comorbidities and risk factors, presumed triggering medications, clinical and laboratory parameters, and clinical outcome data were collected. Clinical factors and laboratory data were assessed from the day of admission. Data were collected in an Excel^®^ (Microsoft Corporation, Redmond, WA, USA) database.

Data were checked for consistency and the normal distribution Fisher’s exact test or Pearson’s test were used to analyze cross-tabulations. Independent *t*-tests and randomization tests based on Monte Carlo simulation with and without the assumption of variance homogeneity, as well as bootstrap logistic regression analysis with odds ratios and 95% CI, were also used. Classification tree analysis based on exhaustive chi-square automatic interaction detection (CHAID method) with 10-fold cross-validation were used to build prediction models for in-house mortality. Sensitivity, specificity, negative and positive predictive values, and total accuracy were computed to estimate the performance of the models. Area under the curve (AUC) of receiver operating characteristic (ROC) curves with 95% CI intervals were used to compare SCORTEN, ABCD-10, Re-SCORTEN, and the classification tree analysis. All reported tests were two-sided, and *p*-values < 0.05 were considered statistically significant. All statistical analyses in this report were performed by use of NCSS (NCSS 2022, NCSS, LLC. Kaysville, UT, USA), STATISTICA 13 (Hill, T. & Lewicki, P. Statistics: Methods and Applications. StatSoft, Tulsa, OK, USA). Categorical variables were reported as total numbers with percentages in parentheses where appropriate. Quantitative variables were reported as mean with standard deviation.

## 3. Results

A total of 68 patients could be included in the study, including 33 survivors and 35 non-survivors. Correspondingly, the mortality rate in the study was 51%. Table 2 provides an overview of the demographic characteristics, clinical and laboratory parameters, and prognostic scores of the two groups. In terms of age, survivors had a mean age of 64.1 years (±18.7), whereas non-survivors were slightly older, with a mean age of 68.5 years (±17.4). This age difference was not statistically significant (*p* = 0.32). With regard to the gender composition of the two groups, 36% of survivors were male compared to 54% of non-survivors (*p* = 0.15).

There were no statistically significant differences between survivors and non-survivors regarding comorbidities and risk factors, including diabetes (*p* = 0.30), peripheral vascular disease (PVD) (*p* = 0.11), obesity (*p* = 1. 00), immunosuppression (*p* = 1. 00), chronic obstructive pulmonary disease (COPD) (*p* = 0.11), heart failure (*p* = 1.00), chronic kidney disease (CKD) (*p* = 0.59), dialysis (*p* = 0.71), human immunodeficiency virus (HIV) infection (*p* = 0.49), cancer (*p* = 0.51), hypertension (*p* = 1.00), alcohol abuse (*p* = 1.00), and drug abuse (*p* = 0.49).

In terms of BSA (at admission), expressed as a percentage, survivors had significantly lower values (43.4% ± 29.7%) than non-survivors (56.8% ± 26.2%, *p* < 0.05). Both groups had an equal proportion of patients with SJS (6% in each group; *p* = 0.95). Among survivors, 36% had SJS/TEN overlap, while only 11% of non-survivors had this condition (*p* = 0.02). Conversely, 58% of survivors had TEN, compared to 83% of non-survivors (*p* = 0.02). Regarding heart rate (≥120 per minute), 12% of survivors and 20% of non-survivors met this criterion, but the difference did not reach statistical significance (*p* = 0.73).

The platelet count showed a significant difference between survivors and non-survivors (160.2 vs. 221.4 per mm^3^). Creatinine levels were also lower in survivors (1.3 ± 1.0 mg/dL) compared to non-survivors (1.9 ± 1.8 mg/dL; *p* = 0.05). Serum blood urea nitrogen (BUN) levels were significantly lower in survivors (30.9 ± 19.7 mmol/L) compared with non-survivors (45.2 ± 30.6 mmol/L, *p* = 0.02). In contrast, other laboratory parameters such as leukocyte count, hemoglobin, red cell distribution width (RDW), RDW/hemoglobin ratio, aspartate transaminase (AST), alanine transaminase (ALT), C-reactive protein (CRP), procalcitonin, glucose, lactate, base excess, and bicarbonate did not show statistically significant differences between the two groups. Among prognostic scores, only Re-SCORTEN showed a statistically significant difference between survivors and non-survivors (3.0 vs. 4.2; *p* = 0.01). SCORTEN (*p* = 0.20) and ABCD-10 (*p* = 0.65) showed no significant differences between the two groups.

In Table 3, we presented the drugs associated with the occurrence of SJS/TEN. In total, among the 68 patients, 26 drugs were identified as being associated with cases of SJS/TEN. Among these, Metamizole was the most frequent causative agent, with eight cases, followed by Allopurinol with seven cases. Additionally, Piperacillin/Tazobactam and Ciprofloxacin were each associated with four cases. In 14 cases, no specific medication could be definitively identified as the trigger. For some patients, two or three medications were considered as potential triggers; these cases have also been included in the table.

**Figure 1:**
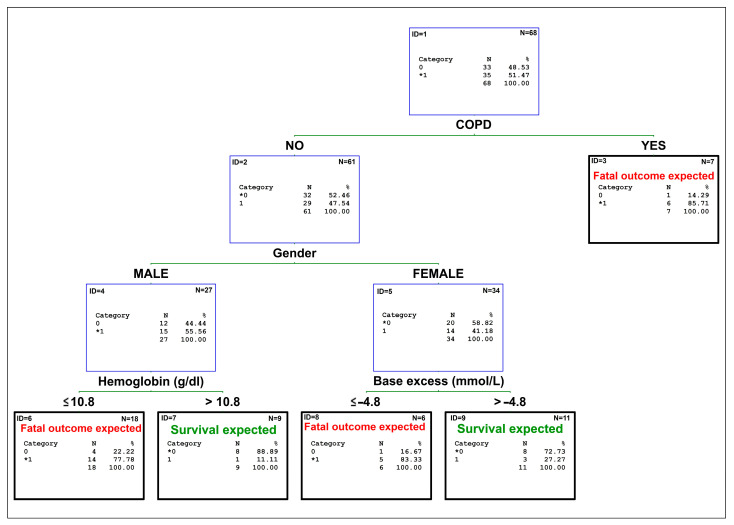
Classification tree model for predicting mortality at admission. Categories marked with an asterisk (*) represent the outcome (0 = survival, 1 = death) that is more likely at that specific decision point, based on the data distribution.

Using our data, we constructed a classification tree to predict outcomes (survival vs. fatal outcome) in patients with SJS/TEN at admission (see Figure 1). The classification tree is based on three levels and includes one comorbidity (COPD), one demographic factor (gender), and two laboratory parameters (hemoglobin and base excess). The classification tree model achieved an accuracy of 89%, a negative predictive value of 81%, a positive predictive value of 95%, a sensitivity of 93%, and a specificity of 86%.

**Figure 2:**
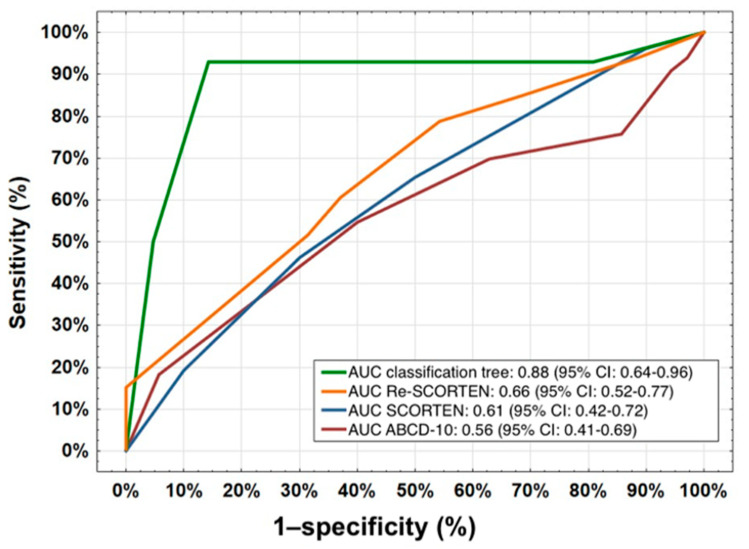
ROC curve analysis of SCORTEN, Re-SCORTEN, ABCD-10, and the classification tree with the respective AUC.

We performed an ROC curve analysis that included SCORTEN, Re-SCORTEN, ABCD-10, and the classification tree (see Figure 2). The classification tree had the highest AUC of 0.88. Of the existing prognostic scores, Re-SCORTEN had the highest AUC of 0.66, followed by SCORTEN with an AUC of 0.61 and ABCD-10 with 0.56.

## 4. Discussion

The findings of our study on SJS and TEN provide valuable insights into the clinical characteristics, prognostic factors, and outcomes of patients affected by these severe drug-induced cutaneous adverse reactions. While several established prognostic scores such as SCORTEN, Re-SCORTEN, and ABCD-10 have been widely used to estimate mortality in SJS/TEN, our results highlight certain limitations of these scores and suggest additional factors that may better predict patient outcomes. Therefore, we designed a classification tree model including these factors.

Our study underscores the significant role of body surface area (BSA) involvement in predicting mortality, as shown by the higher BSA in non-survivors compared to survivors (56.8% vs. 43.4%; *p* = 0.05). This observation is in line with previous studies that consistently demonstrate a strong association between the extent of skin detachment and mortality risk. For instance, a study by Bastuji-Garin et al., which introduced the SCORTEN score, identified BSA as a key determinant of mortality in SJS/TEN patients [10]. Similarly, other studies have confirmed that increasing BSA involvement correlates with worse outcomes [13,14].

Our findings also revealed that certain laboratory parameters, such as creatinine and blood urea nitrogen (BUN), are significantly elevated in non-survivors, indicating renal dysfunction. This aligns with previous studies suggesting that acute kidney injury is a common complication in SJS/TEN and serves as an independent risk factor for mortality [15,16].

Interestingly, our study identified hemoglobin and base excess as significant predictors of mortality, particularly when stratified by gender. These findings are novel and have therefore not been incorporated into existing prognostic scores such as SCORTEN and Re-SCORTEN. Following these findings, we included hemoglobin and base excess in our classification three model.

The role of comorbidities such as chronic obstructive pulmonary disease (COPD) was also highlighted in our classification tree model. While COPD has not been previously emphasized in the SJS/TEN literature, its presence may exacerbate respiratory complications, which are common in severe SJS/TEN cases due to mucosal involvement. Other studies have similarly shown that pre-existing comorbidities, such as recent malignancy, pulmonary disease, and pre-existing severe kidney or liver disorder, increase the risk of mortality [13,16].

The performance of the established prognostic scores in our cohort was poor, with Re-SCORTEN demonstrating the highest AUC (0.66), followed by SCORTEN (0.61) and ABCD-10 (0.56). These findings are consistent with prior studies reporting overestimation of SCORTEN [17].

Our classification tree model achieved an AUC of 0.88, outperforming the existing scores. While this finding is promising, it is important to interpret it cautiously due to the relatively small sample size and the lack of external validation. In addition, it has to be noted that the 95% confidence intervals of the areas under the ROC curves overlap and thus it cannot be proven that the classification tree model is significantly better. Classification tree models offer advantages such as simplicity and clinical interpretability, which make them attractive tools for bedside decision making. However, larger multicenter studies are needed to confirm the generalizability of our model. Future research should also explore advanced machine learning techniques, such as neural networks or support vector machines, to further improve predictive accuracy.

Our analysis of causative drugs revealed Metamizole as the most frequently implicated agent, followed by Allopurinol and antibiotics such as Piperacillin/Tazobactam and Ciprofloxacin. While Allopurinol is a well-documented trigger for SJS/TEN in numerous studies [18,19], the high frequency of Metamizole in our cohort is noteworthy. This finding contrasts with the international literature, where Metamizole is rarely reported as a causative agent [20,21,22]. The high prevalence of Metamizole-related cases in our study likely reflects regional prescribing practices, particularly in Germany, where Metamizole is widely used as an analgesic [23]. This highlights the importance of considering geographic variations in drug utilization when analyzing causative agents of SJS/TEN.

In approximately 20% of cases, no specific causative drug could be identified. This is consistent with other studies, where up to 25% of SJS/TEN cases remain idiopathic despite thorough investigation [24,25]. The inability to pinpoint a causative agent underscores the challenges in establishing drug causality and highlights the need for improved pharmacovigilance systems.

The results of our study provide valuable insights for both clinical practice and future research. While existing prognostic scores such as SCORTEN and Re-SCORTEN remain useful tools, our findings suggest that additional parameters, such as hemoglobin, base excess, and comorbidities like COPD, may enhance mortality prediction in SJS/TEN. The development of the classification tree model represents a step toward personalized risk assessment; however, its clinical utility requires validation in larger, independent cohorts.

We advocate for multicenter studies with larger sample sizes to validate our findings and explore the integration of machine learning techniques for prognostic modeling. Moreover, regional differences in drug triggers, such as the high prevalence of Metamizole in our cohort, should be considered in future studies to better understand the epidemiology of SJS/TEN.

### Limitations

This study has some limitations, including its small sample size and the possibility of other causative drugs not being captured. Better performance of the classification tree-based model compared to the existing prognostic scores cannot be proven following the overlap of 95% confidence intervals.

## 5. Conclusions

This study highlights important clinical and laboratory predictors of mortality in SJS/TEN and identifies limitations of existing prognostic scores. Our classification tree model shows potential predictors of mortality and provides guidance for further research in this field. Furthermore, the tree model could complement existing prognostic scores, improving their predictive quality. Due to the possible over-interpretation of individual factors and the creation of the model based on data from a single center, use in a clinical setting as part of a decision-making process is currently not recommended. Future studies with larger samples are required to validate these results and improve the prediction models for SJS/TEN. Additionally, regional variations in drug triggers should be considered to improve prevention and management strategies for SJS/TEN.

## Figures and Tables

**Table 1:** Prognostic scoring systems with individual criteria for SJS/TEN mortality assessment: SCORTEN by Bastuji-Garin et al. [10], Re-SCORTEN by Koh et al. [12], and ABCD-10 by Noe et al. [11].

SCORTEN	Points	Re-SCORTEN	Points	ABCD-10	Points
Age > 40 years old	1	Age > 40 years old	1	Age > 50 years old	1
Heart rate > 120 beats per minute	1	Heart rate > 120 beats per minute	1	Bicarbonate < 20 mmol/L	1
Heart rate > 120 beats per minute	1	Heart rate > 120 beats per minute	1	Cancer/malignancy	1
Initial epidermal detachment BSA > 10%	1	Initial epidermal detachment BSA > 10%	1	Dialysis prior to admission	1
Serum urea >10 mmol/L	1	Serum urea > 10 mmol/L	1	Initial epidermal detachment BSA ≥ 10%	1
Serum glucose > 14 mmol/L	1	Serum glucose > 14 mmol/L	1		
Bicarbonate < 20 mmol/L	1	Bicarbonate < 20 mmol/L	1		
		RDW/Hb ≥ 1.19	2		
**Score Result**	**Predicted Mortality rate**	**Score Result**	**Predicted Mortality Rate**	**Score Result**	**Predicted Mortality Rate**
0–1	3.2%	0	1.9%	0	2.3%
2	12.1%	1	4.3%	1	5.4%
3	35.3%	2	8.7%	2	12.3%
4	54.3%	3	14.0%	3	25.5%
≥5	90.0%	4	23.0%	4	45.7%
		5	35.3%	5	67.4%
		6	54.5%	6	83.6%
		7	73.2%		
		8–9	86.1%		

Abbreviations: BSA = body surface area; Hb = hemoglobin; RDW = red cell distribution width; Re-SCORTEN = Revised SCORTEN; SCORTEN = Severity-of-Illness Score for TEN; SJS = Stevens–Johnson syndrome; TEN = toxic epidermal necrolysis. Predicted mortality rates are based on the original study descriptions for each scoring system. The score and predicted mortality rate of each scoring system are highlighted by a bold subheading.

**Table 2:** Demographics, comorbidities and risk factors, clinical and laboratory parameters, and prognostic scores of patients (survivors vs. non-survivors).

	Survivors (*n* = 33)	Non-Survivors (*n* = 35)	*p*-Value
**Demographics**			
Age	64.1 ± 18.7	68.5 ± 17.4	0.32
Male	12 (36%)	19 (54%)	0.15
**Comorbidities and risk factors**			
Diabetes	8 (24%)	13 (37%)	0.30
PVD	1 (3%)	6 (17%)	0.11
Obesity	7 (21%)	8 (23%)	1.00
Immunosuppression	2 (6%)	3 (9%)	1.00
COPD	1 (3%)	6 (17%)	0.11
Heart failure	9 (27%)	9 (26%)	1.00
CKD	8 (24%)	11 (31%)	0.59
Dialysis	3 (9%)	5 (14%)	0.71
HIV infection	1 (3%)	0 (0%)	0.49
Cancer	4 (12%)	7 (20%)	0.51
Hypertension	16 (48%)	18 (51%)	1.00
Alcohol abuse	3 (9%)	4 (11%)	1.00
Drug abuse	1 (3%)	0 (0%)	0.49
**Clinical parameters**			
BSA, %	43.4 ± 29.7	56.8 ± 26.2	**0.05**
SJS	2 (6%)	2 (6%)	0.95
SJS/TEN overlap	12 (36%)	4 (11%)	**0.02**
TEN	19 (58%)	29 (83%)	**0.02**
Heart rate (≥120 per min)	4 (12%)	7 (20%)	0.73
**Laboratory parameters**			
Leukocyte count, per mm^3^	10.9 ± 10.5	13.5 ± 11.3	0.34
Hemoglobin, g/dL	11.2 ± 1.7	10.5 ± 2.3	0.11
RDW, %	16.1 ± 2.4	16.3 ± 2.4	0.75
RDW/hemoglobin	2.0 ± 2.8	1.6 ± 0.4	0.49
Platelets, per mm^3^	160.2 ± 91.0	221.4 ± 113.3	**0.02**
Creatinine, mg/dl	1.3 ± 1.0	1.9 ± 1.8	0.05
Serum BUN, mmol/L	30.9 ± 19.7	45.2 ± 30.6	**0.02**
AST, U/L	66.9 ± 87.5	85.9 ± 231.3	0.88
ALT, U/L	61.4 ± 73.5	61.4 ± 144.7	1.00
CRP, mg/dL	17.4 ± 26.5	13.4 ± 9.5	0.52
Procalcitonin, ng/mL	4.1 ± 8.9	3.2 ± 0.6	0.69
Glucose, mg/dL	166.3 ± 52.5	154.6 ± 54.1	0.41
Lactate mmol/L	1.7 ± 0.84	2.0 ± 0.98	0.30
Base excess, mmol/L	0.01 ± 4.2	−0.24 ± 3.8	0.06
Bicarbonate, mmol/L	24.3 ± 3.8	22.9 ± 3.7	0.21
**Prognostic Scores**			
SCORTEN	2.8 ± 1.2	3.3 ± 1.2	0.20
Re-SCORTEN	3.0 ± 2.0	4.2 ± 1.8	**0.01**
ABCD-10	3.0 ± 1.8	3.1 ± 1.4	0.65

Abbreviations: ALT = alanine aminotransferase; AST = aspartate aminotransferase; BSA = body surface area; BUN = blood urea nitrogen; CKD = chronic kidney disease; COPD = chronic obstructive pulmonary disease; CRP = C-reactive protein; HIV = human immunodeficiency virus; PVD = peripheral vascular disease; RDW = red cell distribution width; Re-SCORTEN = Revised SCORTEN; SCORTEN = Severity-of-Illness Score for TEN; SJS = Stevens–Johnson syndrome; TEN = toxic epidermal necrolysis. Continuous data are presented as mean ± standard deviation (SD). *p*-values < 0.05 are considered statistically significant. Key overarching categories and statistically significant *p*-values are highlighted in bold.

**Table 3:** Medications associated with the occurrence of SJS/TEN.

Medication	Frequency
Metamizole	8
Allopurinol	7
Piperacillin/Tazobactam	4
Ciprofloxacin	4
Methotrexate	2
Ibuprofen	2
Cefuroxime	2
Penicillin	2
Diclofenac	2
Azithromycin	2
Amoxicillin/clavulanic acid	2
Moxifloxacin	1
Amoxicillin	1
Aciclovir	1
Amitriptyline	1
Melperone	1
Paclitaxel	1
Apalutamide	1
Oseltamivir	1
Acetylsalicylic acid	1
Sulfonamide	1
Sulfasalazine	1
Pemetrexed	1
Carboplatin	1
Fluconazole	1
Vancomycin	1
No information	14

Abbreviations: SJS = Stevens–Johnson syndrome; TEN = toxic epidermal necrolysis. Drug frequencies are based on confirmed cases in which a causative drug was identified. In 14 cases, no specific medication could be definitively identified as the trigger.

## Data Availability

The data that support the findings of this study are available upon reasonable request from the corresponding author, [D.B.].
